# A randomized controlled study comparing a vessel sealing system with the conventional technique in axillary lymph node dissection for primary breast cancer

**DOI:** 10.1186/s40064-016-2710-7

**Published:** 2016-07-07

**Authors:** Tomoko Seki, Tetsu Hayashida, Maiko Takahashi, Hiromitsu Jinno, Yuko Kitagawa

**Affiliations:** Department of Surgery, Keio University School of Medicine, 35 Shinanomachi, Shinjuku, Tokyo 160-8582 Japan; Department of Surgery, Teikyo University, Tokyo, Japan

**Keywords:** Breast cancer, Vessel sealing system, Axillary lymph node dissection, Randomized controlled trial

## Abstract

**Objective:**

This study aimed to compare the efficacy and safety of the newest bipolar vessel sealing system (BVSS; LigaSure™ Small Jaw) to that of conventional technique in axillary dissection.

**Methods:**

Sixty-one patients with breast cancer were randomized to a conventional dissection surgical technique (CONV group; n = 30) by scalpel and monopolar cautery or that using a vessel sealing system (BVSS group; n = 31).

**Results:**

There was a significant difference between both groups in the mean number of days until drain removal (6.4 ± 2.9 vs. 8.2 ± 3.8 days; *P* value = 0.033), and the mean total volume of drainage fluid (365.3 ± 242.2 vs. 625.1 ± 446.6 mL; *P* value = 0.009). The incidence of seroma was similar in both groups (43.3 vs. 37.9 %; *P* value = 0.673). There was no statistically significant difference in axillary dissection operating time (66 vs. 70 min; *P* value = 0.371), or the mean volume of blood loss (18.2 ± 31.1 vs. 20.6 ± 26.3 mL; *P* value = 0.663).

**Conclusions:**

Our results suggest that BVSS is a more effective device when compared to the conventional techniques in axillary dissection.

## Background

Over the past several decades, there has been a strong trend toward minimizing the extent of surgical resection in the management of patients with breast cancer. Sentinel lymph node biopsy (SLNB) has become the standard procedure for clinical node negative breast cancer surgery, and axillary lymph node dissection (ALND) is required much less frequently than earlier. However, ALND is still the standard procedure when aiming to control local recurrence and classify the tumor stage in patients with advanced breast cancer and multiple lymph node metastases. The number of involved lymph nodes has become an important prognostic factors that guide selection of those patients who might benefit from adjuvant treatment (Clarke et al. 2005; Orr 1999; Kodama et al. 2006; Fisher et al. 1985, 2002).

ALND is associated with postsurgical complications, such as lymphorrhea, seroma formation, lymphoedema and limited range of motion of the shoulder. Lymphorrhea occurrence and seroma formation is associated with delayed drain removal, prolonged in-hospital stay, and delayed adjuvant therapy. In the literature (Burak et al. 1997; Cortadellas et al. 2011; Gonzalez et al. 2003; Hashemi et al. 2004; Agrawal et al. 2006), frequency of seroma formation is reportedly 2.5–85 %. It has been suggested that efficient methods of sealing blood and lymph vessels during ALND may play a key role in reducing postoperative lymphorrhea. Several new surgical devices, such as a harmonic scalpel and bipolar vessel sealing system (BVSS), have been proposed. Several randomized controlled trials comparing harmonic scalpels or BVSS to the conventional technique have reported on the efficacy of harmonic scalpels and BVSS, however, some trials concluded that there is no significant difference between these surgical devices (Burdette et al. 2011; Currie et al. 2012; He et al. 2012; Lumachi et al. 2013; Iovino et al. 2012).

The LigaSure Small Jaw (LSJ; Covidien, Energy-Based Devices, Boulder, Colorado) is the newest BVSS in the LigaSure family, introduced in 2010. This device has features like a small curved jaw, low-temperature profile, minimal thermal spread, and multifunctionality. There has been no prospective study comparing the LSJ with conventional surgical methods in ALND. In this study, we have conducted a prospective randomized trial comparing surgical results on using the LSJ in ALND for breast cancer.

## Patients and methods

### Study population

This study was designed as a prospective, open, single center, single blind randomized controlled trial. Study patients were recruited and underwent surgery in the surgical department of the Keio University Hospital. Patients were enrolled between May 2013 and June 2015 and provided written informed consent before participating in the trial. Patients were randomly assigned to the study group (BVSS group) or the control group (conventional treatment; CONV group) with a 1:1 ratio by using covariate adapting randomization. In the literature, several risk factors have been identified for seroma and lymphorrhea: age greater than 60 years, elevated body mass index (BMI), tumor size, neoadjuvant chemotherapy, extent of gland resection, and number of metastatic lymph nodes. The patients were randomly assigned (1:1) to the BVSS or conventional group and balanced according to age, BMI, type of surgery (i.e., breast conserving surgery or total mastectomy) and experience of surgeon (less or more 7 years). The investigating surgeons were informed of the treatment allocation via the randomization software before they performed the procedures. The patients as well as the nurses who measured the daily drainage volumes were blinded to the group assignment. The BVSS group consisted of 31 patients who underwent ALND with the LigaSure Small jaw; the CONV group consisted of 30 patients who underwent ALND with conventional devices such as mono or bipolar electrocautery.

Preoperative data such as breast cancer staging, age, and BMI were obtained for all patients. Several basic criteria had to be met before patients were included in the study: (1) cytological proof of breast cancer, (2) curative surgery (mastectomy or breast-conserving surgery), (3) clinical N0 (no palpable axillary nodes) and confirmed to node positive via sentinel node biopsy, (4) N1 (metastasis in movable ipsilateral axillary lymph node(s)) and N2 (metastasis in fixed ipsilateral axillary lymph node(s) or in clinically apparent ipsilateral internal mammary lymph node(s) in the absence of clinically evident axillary lymph node metastasis) according to the 2009 UICC stage classification, 7th edition (UICC, 2009), (4) performance status 0–2 (ECOG scale) and (5) age over 20 years, and (6) patients who provided written informed consent. The exclusion criteria were: (1) a concomitant malignant disease, (2) prior surgery or radiotherapy for axilla and (3) patients with severe diabetes, infections, bleeding diatheses, or taking anticoagulant medication (e.g., aspirin and warfarin).

The study protocol was approved by the Ethical Committee of Keio University Hospital. The trial was registered at University hospital Medical Information Network (UMIN) Clinical Trial Registry with the ID number UMIN000010637.

### Surgical technique and axillary drain

In all patients axillary lymphadenectomy was performed via standard level I and II en-bloc node dissection. The procedure was performed by a specialized breast surgical team, consisted of three senior breast surgeons and four junior breast surgeons in training. The senior surgeons supervised all the procedure of the surgery and postoperative management. Patients randomized to the BVSS group were operated using the LigaSure Small Jaw to seal the lymph and vessels; dissection with monopolar electrocautery or scalpel was limited as much as possible. Patients randomized to the CONV group were operated using scissors/scalpel and mono or bipolar electrocautery or suture ligation to achieve vessel sealing. The control of bleeding was achieved before a suction drain was introduced in the axilla. At the end of the surgery, a closed suction drain (size: 5 mm) was placed in the axilla. In the case of total mastectomy, a closed suction drain (size: 3 mm) was also placed in anterior chest. In all cases, the axillary dissection followed the breast surgery, and surgical time was recorded from the beginning of the axillary dissection to the end of surgery.

At the end of surgery, a standard, noncompressive dressing was applied to all patients. No limitation to arm movement was scheduled. According to protocol, the axillary drain was removed when the daily output was less than 30 mL in the previous 24 h. The total amount of drainage was measured daily during the hospital stay by the nursing staff, who were blinded to the devices used.

Patients were followed-up in out-patients clinic every one or 2 weeks for a minimum of 30 days after hospital discharge.

### Primary and secondary end points

The primary end point was to compare the days until drain removal on using the two different surgical techniques. The secondary end points were to compare the total volume of fluid collected in the axillary drain, surgical time, and the incidence of seroma in both groups. Safety of the experimental technique was judged by the occurrence of postoperative complications and intraoperative blood losses.

A sample size of 60 participants was calculated from our retrospective data before conducting this study. Assuming a difference in drainage volume between two groups with 90 % power and two-sided type I error of 5 %, we required 60 patients with a drop out of 5 %. The mean days until drain removal after the axillary dissection using vessel sealing system versus electrocautery was 6 versus 9 days (*P* = 0.208).

### Statistical analysis

In all patients, the following data were recorded: age, BMI, type of surgery, previous neoadjuvant therapy, duration of operation, intraoperative blood loss measured as the amount of blood aspirated from the operative field and surgical gauzes (10 % blood-soaked gauge pieces weight), duration of drain placement, amount of drained fluid, length of hospital stay, number of punctures of axillary seromas in the outpatient clinics, histological type and immunohistochemical characteristics of the tumor, total number of lymph nodes excised, positive lymph nodes, and early postoperative complications (hematoma, wound infection, wound dehiscence, blood transfusion, and need for readmission). Continuous data were described according to median and range, and comparisons were performed by using the nonparametric Mann–Whitney U test. Categorical variables were described according to the percentages of subjects falling in each category, and analyzed by using the Chi square test. The hazard risk (HR) and the 95 % confidence interval (CI) were also obtained. An error level of *P* < 0.05 was considered statistically significant. The proportion of patients who reached 30 mL of daily output and had the drainage removed in time was described by using the Kaplan–Meier estimate and compared by using the log-rank test. To examine the potential interactions between outcomes and patients’ background, subgroup analysis was performed.

Statistical analysis of the data was performed by using SPSS^®^ Version 20 (SPSS-IBM Inc., Chicago, IL, USA). A value of *P* < 0.05 was considered statistically significant, and all tests were 2-sided. This trial is reported in accordance with the CONSORT statement.

## Results

### Background characteristics of the patients

**Fig. 1:**
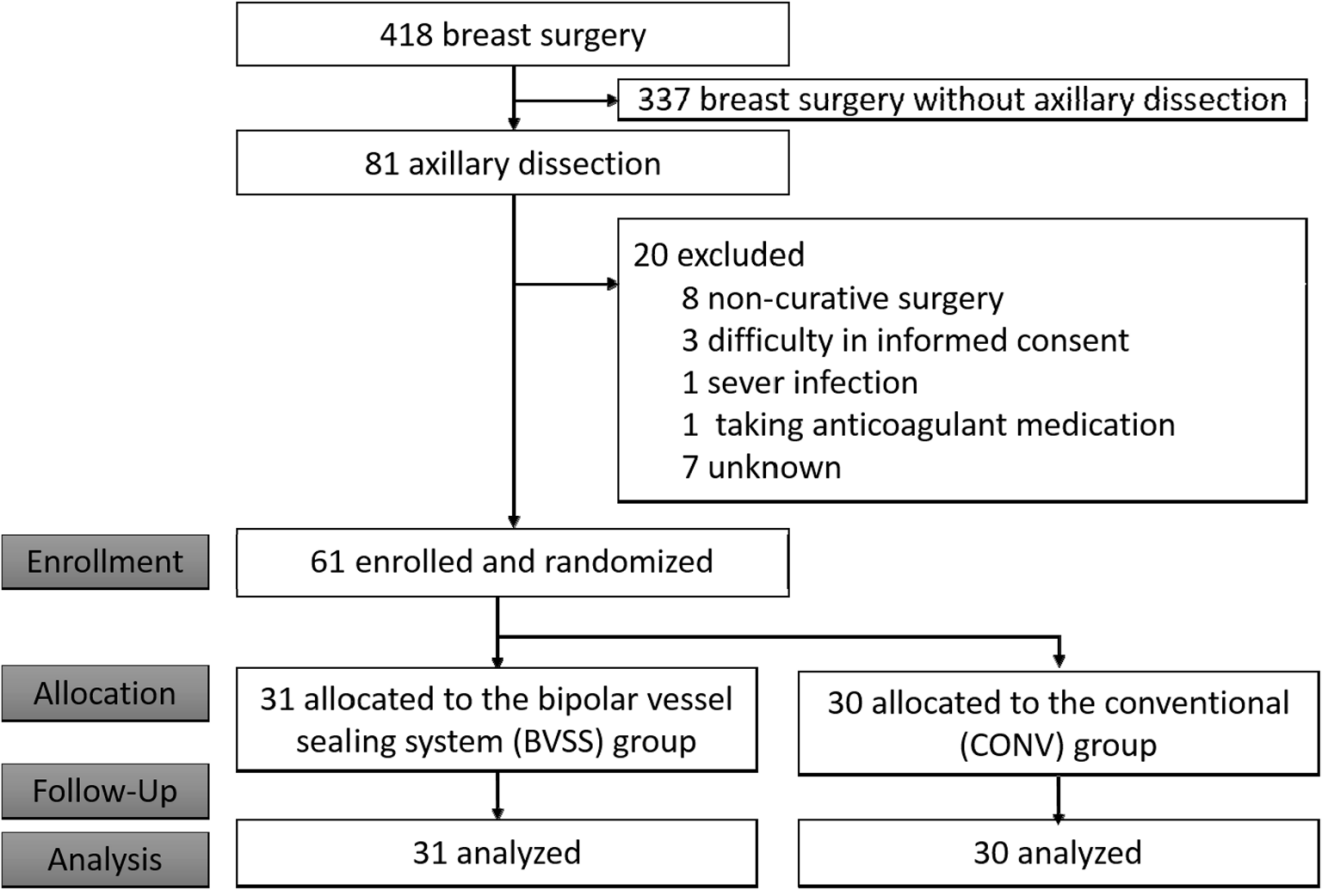
Trial profile

During the study period, 81 patients underwent axillary lymph node dissection out of a total of 418 breast cancer patients. Among these, 61 patients were enrolled and randomized, with 31 in the BVSS group and 30 in the CONV group. No patient was excluded after randomization, and no one was lost to follow up. Finally, a total of 31 patients in the BVSS group, and 30 patients in the CONV group were analyzed on an intention-to-treat basis (Fig. 1). The baseline characteristics of the patients are summarized in Table 1. There were no statistically significant differences between the two groups, except for the HER2 status.

**Table 1:** Characteristics of patients

Variables	Vessel sealing system (BVSS)	Conventional devices (CONV)	*P* value
Number of patients	31	30	
Age at diagnosis (y/o)	59.1 ± 15.6	57.6 ± 11.2	0.663
BMI	21.8 ± 4.0	21.5 ± 3.1	0.731
Comorbidities			
HTN	5 (16.1 %)	2 (6.7 %)	0.246
DM	2 (6.5 %)	1 (3.3 %)	0.573
Smoking	11 (35.5 %)	4 (13.3 %)	0.045
Clinical TNM stage			0.055
0	0 (0.0 %)	1 (3.3 %)	
1	8 (26.7 %)	1 (3.3 %)	
2	18 (60.0 %)	21 (70.0 %)	
3	4 (13.3 %)	7 (23.3 %)	
Clinical T stage			0.111
Tis	0 (0.0 %)	1 (3.3 %)	
T1	11 (35.5 %)	3 (10.0 %)	
T2	16 (51.6 %)	20 (66.7 %)	
T3	1 (3.2 %)	2 (6.7 %)	
T4	3 (9.7 %)	4 (13.3 %)	
Clinical N stage			0.73
N0	11 (35.5 %)	9 (30.0 %)	
N1	17 (54.8 %)	19 (63.3 %)	
N2	1 (3.2 %)	0 (0.0 %)	
N3	2 (6.5 %)	2 (6.7 %)	
Neoadjuvant chemotherapy	21 (67.7 %)	24 (80.0 %)	0.277
Receptor status			
Hormone receptor positive	19 (61.3 %)	12 (40.0 %)	0.286
HER2 positive	4 (13.3 %)	10 (24.1 %)	0.047
Histology			0.331
Ductal carcinoma	31 (100 %)	28 (96.5 %)	
Lobular carcinoma	0 (0.0 %)	1 (3.4 %)	
Others	0 (0.0 %)	1 (3.4 %)	
Nuclear grade			0.679
1	11 (35.5 %)	13 (43.3 %)	
2	10 (32.3 %)	7 (23.3 %)	
3	7 (22.6 %)	5 (16.7 %)	
Unknown	3 (9.7 %)	5 (16.7 %)	
Type of surgery			0.394
Total mastectomy	18 (58.1 %)	18 (60.0 %)	
Breast conserving surgery	12 (38.7 %)	12 (40.0 %)	
Total number of removed lymph nodes	20.3 ± 7.4	18.4 ± 6.7	0.268
Pathological tumor size	2.1 ± 1.6	1.7 ± 1.3	0.290
Total number of pathologically			
Positive lymph nodes			0.273
0	9 (29.0 %)	10 (33.3 %)	
1	7 (22.6 %)	10 (33.3 %)	
2	7 (22.6 %)	4 (13.3 %)	
3	1 (3.2 %)	4 (13.3 %)	
>4	7 (22.6 %)	2 (6.6 %)	
Lymphovascular invasion	16 (51.6 %)	12 (40.0 %)	0.363
Mean days until drain removal	6.4 ± 2.9	8.2 ± 3.8	0.033
Total drain volume (mL)	365.3 ± 242.2	625.1 ± 446.6	0.009
Total operating times (minutes)	180.9 ± 65.5	168.6 ± 30.3	0.670
Axillary dissection operating time (minutes)	65.6 ± 19.6	70.3 ± 21.6	0.371
Estimated blood loss (mL)	18.2 ± 31.1	20.6 ± 26.3	0.663
Postoperative complications			0.673
Postoperative bleeding	0 (0.0 %)	1 (3.3 %)	
Hematoma	0 (0.0 %)	1 (3.3 %)	
Seroma	13 (43.3 %)	11 (37.9 %)	
Wound infection	0 (0.0 %)	0 (0.0 %)	
Flap necrosis	0 (0.0 %)	0 (0.0 %)	
Skin burn	0 (0.0 %)	0 (0.0 %)	
Postoperative hospital stay (days)	8.8 ± 3.1	10.1 ± 3.1	0.077

### Intraoperative data

Overall, 36 (59.0 %) patients underwent total mastectomy, while 24 (39.3 %) patients underwent breast-conserving surgery (partial mastectomy), according to the tumor stage. One patient who was diagnosed with accessory breast cancer underwent axillary dissection only. There was no significant difference in type of surgery between the two groups. There was no significant statistical difference in the axillary dissection operating time (66 vs. 70 min; *P* value = 0.371), or the mean volume of blood loss (18.2 ± 31.1 vs. 20.6 ± 26.3 mL; *P* value = 0.663).

### Postoperative data

**Fig. 2:**
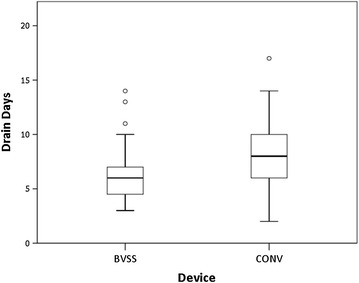
Distribution of the drain days. There was a significant difference between the two groups in the mean number of days until drain removal (6.4 ± 2.9 vs. 8.2 ± 3.8 days; *P* value = 0.033). *Boxplot legend*: *upper horizontal line of box* 75th percentile, *lower horizontal line of box* 25th percentile, *horizontal bar within box* median value and *vertical dotted line* minimum–maximum value. *BVSS* bipolar vessel sealing system, *CONV* conventional devices

**Fig. 3:**
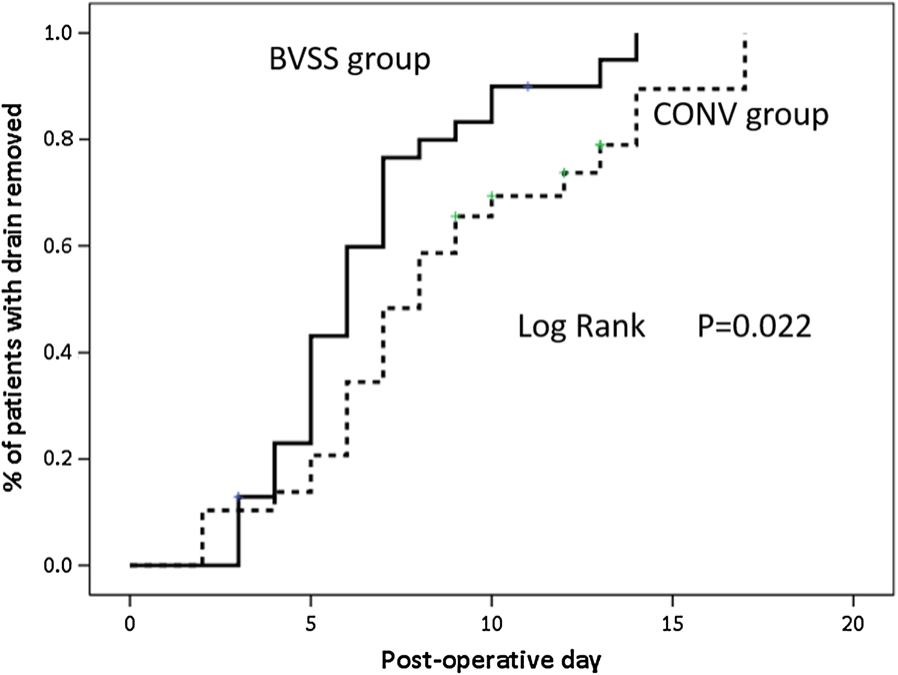
Cumulative percentage of patients with the drain removed over time. The proportion of patients who reached 30 mL of daily output and had the drain removed in time was described by using the Kaplan–Meier estimate and compared by using the log-rank test. Drain days were significantly reduced in the BVSS group (*P* = 0.022). *BVSS* bipolar vessel sealing system, *CONV* conventional devices

**Fig. 4:**
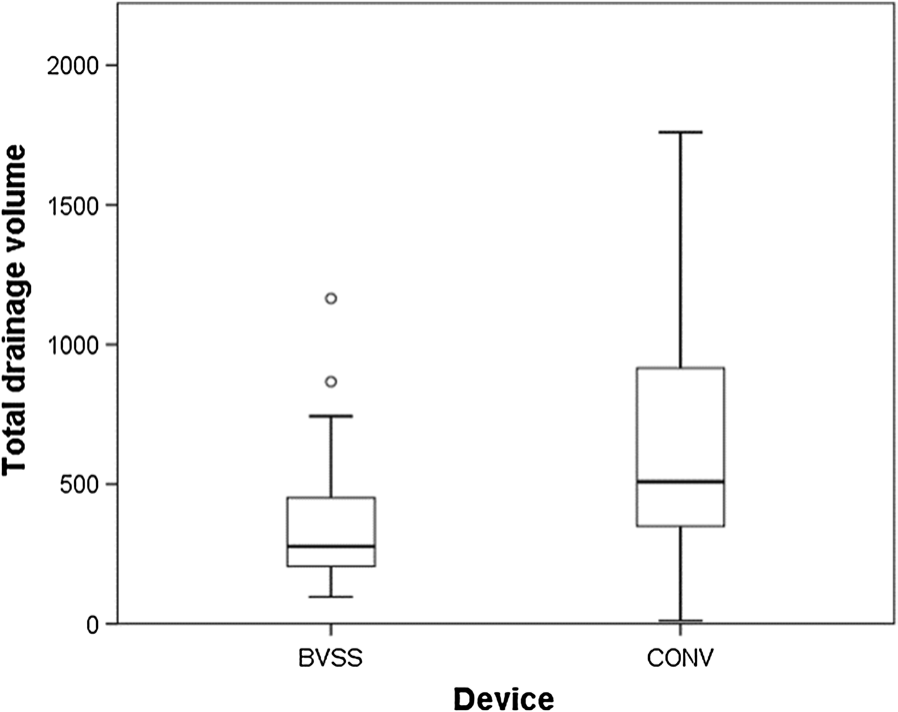
Distribution of the total drainage volume. There was a significant difference between the two groups in the mean total volume of drainage fluid (365.3 ± 242.2 vs. 625.1 ± 446.6 mL; *P* value = 0.009). *Boxplot legend*: *upper horizontal line of box* 75th percentile, *lower horizontal line of box* 25th percentile, *horizontal bar within box* median value and *vertical dotted line* minimum–maximum value. *BVSS* bipolar vessel sealing system, *CONV* conventional devices

The postoperative data is shown in Figs. 2, 3, 4. As shown in Fig. 2, there was a significant difference between the two groups in mean number of days until removal of the drain (6.4 ± 2.9 vs. 8.2 ± 3.8 days; *P* value = 0.033). The proportion of patients who reached 30 mL of daily output and had the drain removed in time was described by using the Kaplan–Meier estimate and compared by using the log-rank test (Fig. 3). The days until drain removal were significantly reduced in the BVSS group (*P* value = 0.022). The mean total volume of drainage was also significantly reduced in the BVSS group (365.3 ± 242.2 vs. 625.1 ± 446.6 mL; *P* value = 0.009) (Fig. 4). The mean number days of postoperative hospital stay was not significantly reduced in the BVSS group (8.8 ± 3.1 vs. 10.1 ± 3.1 days; *P* value = 0.077).

### Complications

Short-term postoperative complications (i.e., bleeding and hematoma) were observed in 1 (3.3 %) patients in the conventional group. None of the patients in either group developed wound infections or experienced skin burns or necrosis. Twenty-four (39.3 %) patients developed an axillary seroma: 13 (43.3 %) and 11 (37.9 %) in the BVSS and CONV groups, respectively (*P* value = 0.673).

### Subgroup analysis

**Fig. 5:**
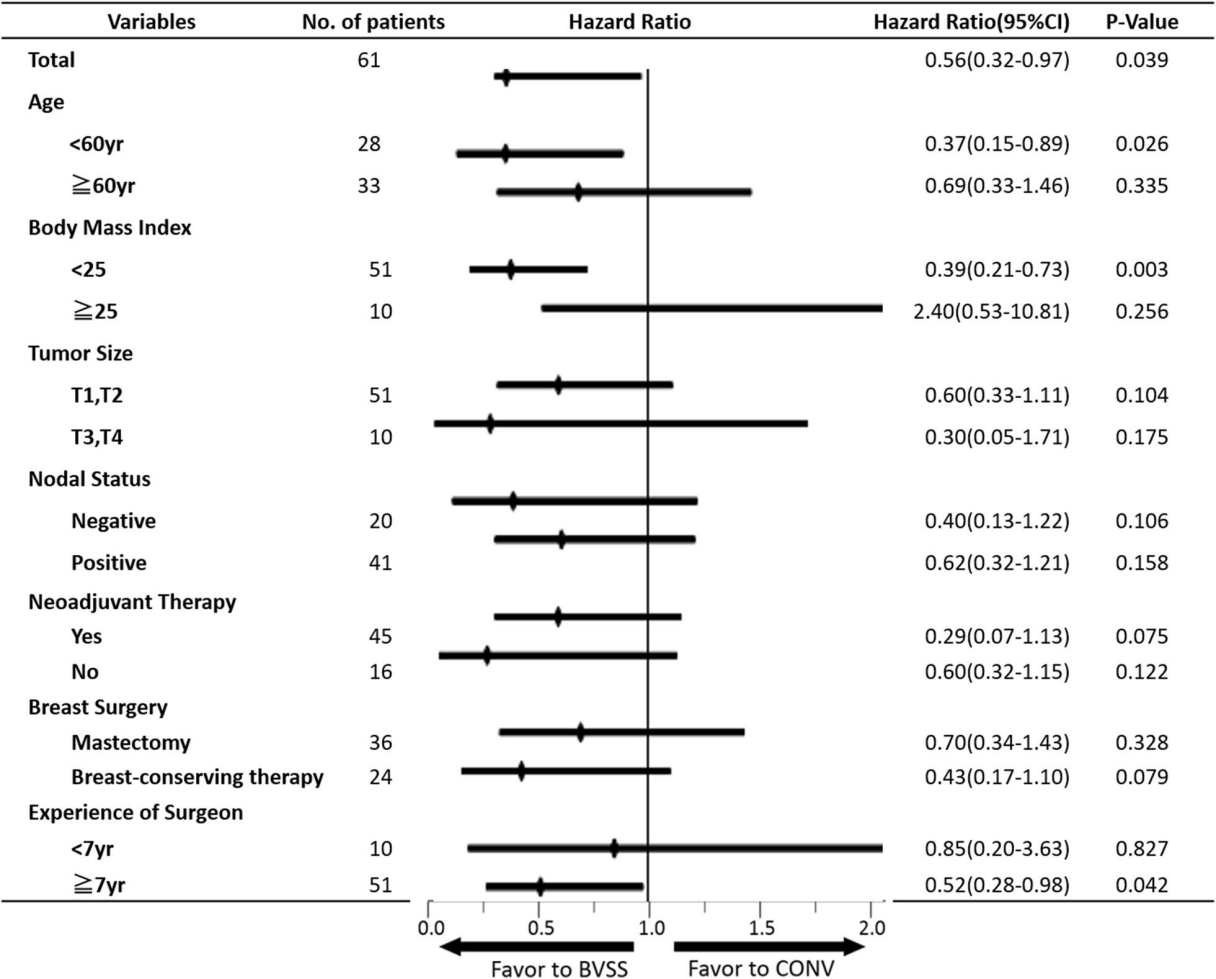
Subgroup analysis of drain days. Subgroup analysis was performed by using the Cox hazard ratio analysis. Hazard ratio and 95 % confidence interval was obtained. Drain days were reduced in the patients with age under 60 years, body mass index less than 25, and patients whose surgery was performed by the surgeon with more than 7 years of experience. *BVSS* bipolar vessel sealing system, *CONV* conventional devices

We performed a subgroup analysis to identify potential interactions between drain days and patients’ background factors (Fig. 5), and between drainage volume and patients’ background factors (Table 2). A significant risk reduction for the days until drain removal was noted in the BVSS group compared to the CONV group in patients with age under 60 years, BMI less than 25, and patients whose surgeries were performed by the surgeon with more than 7 years of experience. A significant drainage volume reduction was also noted in the BVSS group compared to the CONV group in these same patients.

**Table 2:** Subgroup analysis regarding the total drainage volume

Variables	Vessel sealing system (BVSS)	Conventional devices (CONV)	*P* value
Total	365 ± 242	625 ± 447	0.006
Age			
<60 years	293 ± 144	612 ± 438	0.02
≧60 years	425 ± 291	637 ± 468	0.146
BMI			
<25	304 ± 179	594 ± 415	0.005
≧25	620 ± 317	828 ± 657	0.517
Tumor size			
T1, T2	388 ± 330	648 ± 438	0.011
T3, T4	211 ± 107	534 ± 511	0.476
Nodal status			
Negative	410 ± 295	774 ± 475	0.175
Positive	341 ± 212	561 ± 430	0.046
Neoadjuvant therapy			
Yes	374 ± 310	868 ± 511	0.011
No	361 ± 211	564 ± 419	0.092
Breast surgery			
Mastectomy	346 ± 180	505 ± 396	0.265
Breast-conserving therapy	412 ± 321	805 ± 473	0.02
Experience of surgeon			
<7 years	530 ± 441	691 ± 558	0.642
≧7 years	341 ± 201	608 ± 427	0.007

## Discussion

An advantage of using the vessel sealing system is that this procedure does not require direct exposure of blood vessels, which can increase operative time and cause unnecessary bleeding. Previous randomized studies comparing the vessel sealing system with conventional devices have reported that vessel sealing system seems to reduce the drainage volume and shorten the postoperative hospital days (Cortadellas et al. 2011; Nespoli et al. 2012; Tukenmez et al. 2014). The vessel sealing system used in these studies was the LigaSure Precise, not the newest device, the LigaSure Small Jaw.

The LigaSure Small Jaw is a manual device, 18.8 cm long. It is designed to be used in confined surgical spaces where access and visibility are limited. This instrument, designed for open surgery, offers the ability to selectively cut or grasp tissue and permanently seal vessels with a diameter of up to and including 7 mm, lymphatic vessels, and tissue bundles without using sutures, staples, or clips. Although some studies (Yoshimoto et al. 2014; Hwang et al. 2014; Coiro et al. 2015) comparing the LigaSure Small Jaw with conventional suture ligation in thyroidectomy and hepatic resection showed intraoperative blood loss to be statistically significantly less for the LigaSure Small Jaw, this device had not yet been evaluated in axillary dissection. To the best of our knowledge, our study is the first randomized controlled study comparing the LigaSure Small Jaw and conventional devices in axillary dissection. Our study shows that the use of the LigaSure Small Jaw reduced the days until drain removal and postoperative drainage volume when comparing to the conventional devices. It is extremely important to shorten drain days and hospital stay, because delays in these factors can lead to delays in adjuvant therapy. In this study, after subgroup analysis, the vessel sealing system seems to be more effective in the patients with age under 60 years, and BMI less than 25. However this was not conclusive because of the limited sample size.

Although drainage days were reduced on using the vessel sealing system, seroma formation could not be completely avoided. The optimal way to prevent and treat seromas remains inconclusive. Other complications such as postoperative bleeding and skin flap necrosis were not observed when the vessel sealing system was used. No statistically significant difference was seen in intraoperative blood loss on use of the vessel sealing system compared to use of conventional devices. We can conclude that the vessel sealing system is safe to use in axillary dissection. On the other hand, a major criticism of the vessel sealing system seems to be its high cost. However, when taking the discomfort of postoperative drain days into consideration, the device actually might be cost-effective for patients.

This is a randomized controlled study with a well-planned consistent protocol of peri-operative care, data management, and statistical analysis. Patients were strictly followed up until the end of the study. Although randomized controlled studies are usually the highest level of evidence for judging the efficacy of therapeutic interventions, a limitation of this study is that it is not possible to blind the operating surgeon on the result of randomization which could influence the outcome of the study. Another limitation is the low patient population in this study. This study was conducted in a single institution and was therefore not adequately powered to assess the benefit of treatment in different subgroups.

In conclusion, this study suggested the usefulness of the vessel sealing system in axillary dissection. This device reduces the duration until drain removal and total drainage volume, and the complication rates were comparable to those seen on using conventional devices.
